# Phosphorylation of a malate transporter promotes malate excretion and reduces cadmium uptake in apple

**DOI:** 10.1093/jxb/eraa121

**Published:** 2020-03-09

**Authors:** Qi-Jun Ma, Mei-Hong Sun, Jing Lu, Da-Gang Hu, Hui Kang, Chun-Xiang You, Yu-Jin Hao

**Affiliations:** 1 National Key Laboratory of Crop Biology, MOA Key Laboratory of Horticultural Crop Biology and Germplasm Innovation in Huanghuai Region, College of Horticulture Science and Engineering, Shandong Agricultural University, Tai-An, Shandong, China; 2 Department of Plant Biology, Department of Horticulture, Michigan State University, East Lansing, MI, USA; 3 Development Center of Plant Germplasm Resources, College of Life Sciences, Shanghai Normal University, Shanghai, China; 4 University of Warwick, UK

**Keywords:** ALMT14, apple, cadmium, heavy metal, malic acid, SOS2L1

## Abstract

Heavy metal contamination is a major environmental and human health hazard in many areas of the world. Organic acids sequester heavy metals and protect plant roots from the effects of toxicity; however, it is largely unknown how these acids are regulated in response to heavy metal stress. Here, protein kinase SOS2L1 from apple was functionally characterized. MdSOS2L1 was found to be involved in the regulation of malate excretion, and to inhibit cadmium uptake into roots. Using the DUAL membrane system in a screen of an apple cDNA library with MdSOS2L1 as bait, a malate transporter, MdALMT14, was identified as an interactor. Bimolecular fluorescence complementation, pull-down, and co-immunoprecipitation assays further indicated the interaction of the two proteins. Transgenic analyses showed that MdSOS2L1 is required for cadmium-induced phosphorylation at the Ser^358^ site of MdALMT14, a modification that enhanced the stability of the MdALMT14 protein. MdSOS2L1 was also shown to enhance cadmium tolerance in an MdALMT14-dependent manner. This study sheds light on the roles of the MdSOS2L1–MdALMT14 complex in physiological responses to cadmium toxicity.

## Teaser

Protein kinase SOS2L1 phosphorylates the malate transporter ALMT14, resulting in malate excretion from root to rhizosphere to improve cadmium tolerance in apple.

## Introduction

Various human activities, including manufacturing, mining, petroleum refining, and chemical production, lead to heavy metal contamination, which has been identified as a major environmental and human health risk factor (Misra and Pandey, 2005). Cadmium (Cd), one of the most toxic heavy metals, is taken up by plants, where it accumulates in different organs (Broadley *et al.*, 2001; Lux *et al.*, 2011). Cd enrichment in soil occurs from both natural and anthropogenic sources (Pan *et al.*, 2016). Cd concentrations of uncontaminated soils are usually below 0.5 mg kg^–1^, but can reach up to 16.7 mg kg^–1^ depending on the soil parent materials (Vahter *et al.*, 1991; Khan *et al.*, 2017). High Cd concentrations can have toxic effects on soil organisms and Cd can easily transfer into plants and ultimately enter the food chain (Wahid *et al.*, 2009; Li *et al.*, 2016; Zhou *et al.*, 2016).

Metal toxicity includes alteration of speed of cell proliferation and organization of cellular structures underlying cell proliferation, as well as programmed cell death and/or necrosis (Krzesłowska, 2011). Many kinds of toxic metals affect plant microtubules, which are highly dynamic components of plant morphogenesis (Horiunova *et al.*, 2016). These metals can affect photosynthesis, alter protein synthesis, inhibit enzyme activity, disrupt the transport and movement of essential ions, inhibit stoma function, generate oxidative stress, and severely affect growth (Horiunova *et al.*, 2016). In addition, Cd accumulation in edible organs can be a significant factor in food quality and safety (Lux *et al.*, 2011; Luo *et al.*, 2018).

Plants have evolved two main strategies to limit Cd stress: internal tolerance and external avoidance (Baker and Proctor, 1990). The cell wall is the first structure of plant cells that sequesters heavy metals. At the same time, its synthesis and composition can be severely affected by Cd stress (Parrotta *et al.*, 2015). Molecular and physiological strategies of internal tolerance have been well studied, especially with regard to transport, distribution and translocation of Cd. Several transporters are known to be involved in these processes, including the heavy metal P1B-ATPase AtHMA4, which is a member of the P-type metal ATPase family, and functions as a Zn/Cd transporter for loading Cd into the xylem in Arabidopsis (Mills *et al.*, 2005). In addition, a defensin-like protein, CAL1, can chelate Cd and facilitate its secretion from xylem parenchyma cells into the xylem vessels, thereby decreasing Cd accumulation in the cytosol and promoting Cd translocation from roots to shoots (Luo *et al.*, 2018). Other strategies to limit Cd damage include increasing translocation to the apoplast or aerial portions of the plant (Ueno *et al.*, 2010; Uraguchi & Fujiwara, 2012). It is also known from studies of many plant species that tolerance of Cd is related to its accumulation in the vacuoles (Cosio *et al.*, 2005; Korenkov *et al.*, 2007). Cd is translocated across the tonoplast by H^+^/Cd^2+^-antiporters, such as AtCAX2 and AtCAX4 (Chen *et al.*, 2016), and heavy metal P1B-ATPases, while Cd-chelates are transported by ABC transporters, including AtMRP3 (Shim *et al.*, 2009; Park *et al.*, 2012). Within plant cells, Cd is bound to S-containing ligands, such as phytochelatins (PCs), glutathione (GSH), and metallothioneins (MT), and Cd is often sequestered in the vacuole as Cd–phytochelatin complexes (Fenik *et al.*, 2007; DalCorso *et al.*, 2008; Ismael *et al.*, 2019).

Plants can also limit damage from external Cd by reducing its bioavailability, and thereby reducing uptake, through the production of ligands that are capable of complexation or chelation with heavy metal ions in soils and water (Dong *et al.*, 2007). For example, approximately half of all photosynthates are transported to the roots, and of this portion approximately 12–40% can be released as exudates into the rhizosphere, the interface between roots and the soil (Hinsinger *et al.*, 2006; Kushwaha *et al.*, 2015). Root secretion includes the release of organic ligands (e.g. carbohydrates, organic acids, humic acids, polypeptides, proteins, amino acids, nucleic acids, etc.) and inorganic ligands (Cl^−^, SO_4_^2−^, NH_4_^+^, CO_3_^2−^, and PO_4_^3−^). These substances function not only as energy sources for microorganisms but also as ligands that can reduce the toxicity of heavy metals through chelation and sequestration (McGrath *et al.*, 2001; Chen *et al.*, 2016). Small peptides, organic acids, and amino acids can bind free and cytosolic metal ions, resulting in metal detoxification, and this may affect their solubility and phytoavailability (Chen *et al.*, 2011). Specific examples of species whose roots excrete organic acids (e.g. oxalic acid, malic acid, and citric acid) that chelate Cd^2+^ and prevent its uptake by roots are wheat and buckwheat (*Fagopyrum esculentum*) (Dong *et al.*, 2007; Guo *et al.*, 2016).

Numerous genes and proteins are involved in responses to Cd stress. In the heavy metal-accumulating plants *Lunularia cruciata* and *Brassica juncea*, gene expression patterns were shown to change in response to Cd treatment (Zhang *et al.*, 2012), and the expression of protein kinases, such as mitogen-activated protein kinases (MAPKs), is also activated upon exposure to excessive Cd in the roots of alfalfa seedlings (Liu *et al.*, 2010). However, it is not clear whether and how these kinases directly regulate heavy metal-associated genes.

Another type of protein kinase related to metal ion responses is represented by the Calcineurin B-Like (CBL)-interacting protein kinases (CIPKs) (Chen *et al.*, 2016). CIPK24 (also known as SOS2), a Ser/Thr protein kinase, and SOS3 (also known as CBL4), a Ca^2+^-binding protein, are both able to respond to salt-induced calcium signals (Zhu, 2003). SOS2 not only regulates Na^+^/H^+^ antiporter SOS1 transport activity, but also regulates the activity of other transporters, such as the NHX tonoplast Na^+^/H^+^ antiporters. These antiporters are activated through a mechanism related to the Ca^2+^ sensor AtCBL10, and promote the sequestration of excess intracellular Na^+^ in the vacuole (Mahajan *et al.*, 2008). An interaction between SOS2 and an unknown Ca^2+^ sensor allows the vacuolar membrane-localized H^+^/Ca^2+^ antiporter CAX1 to control intracellular Ca^2+^ homeostasis (Cheng *et al.*, 2005), and SOS2 is thought to regulate the activity of AtHKT1, leading to Na^+^ entry into root cells under salt stress in Arabidopsis (Uozumi *et al.*, 2000; Zhu, 2003; Mahajan and Tuteja, 2005). Finally, SOS2 physically interacts with the V-ATPase regulatory subunits VHA-B1 and VHA-B2 in Arabidopsis to regulate V-ATPase activity required for ion transport during salt stress (Batelli *et al.*, 2007). However, it is not known whether and how CIPK proteins respond to Cd stress.

MdSOS2L1 is an apple SOS2 homolog that interacts with and phosphorylates the vacuolar V-ATPase regulatory subunit MdVHA-B1, thereby modulating malate accumulation in the vacuoles, and its ectopic or overexpression enhances salt tolerance in transgenic plants (Hu *et al.*, 2016a). In the present study, MdSOS2L1 was functionally characterized and shown to inhibit Cd uptake by roots by regulating malate excretion. The potential utilization of MdSOS2L2 and its interacting protein in developing novel strategies or techniques for cultivation and breeding to avoid Cd uptake in fruit trees and other crops is discussed.

## Materials and methods

### Plant materials, growth conditions, and CdCl_2_ treatments

Tissue cultures of *Malus×domestic* cv. ‘Royal Gala’ apples were used as the wild type (WT). Transgenic ‘Royal Gala’ apple plants overexpressing MdSOS2L1 and MdALMT14 as well as the non-transformed control were grown on Murashige and Skoog (MS) medium supplemented with 1.0 mg l^−1^ naphthyl acetate (NAA) and 0.5 mg l^−1^ 6-benzylaminopurine (6-BA) at 25 °C under long-day conditions (16 h light/8 h dark). The plants were subcultured at 30 d intervals. For CdCl_2_ treatment, apple shoot cultures were cultivated on MS medium supplemented with 0.5 mg l^−1^ indole-3-acetic acid (IAA), 1.5 mg l^−1^ 6-BA, and 100 µM CdCl_2_.

The apple calli used were induced from young embryos of ‘Orin’ apples (*Malus domestica* Borkh.) and subsequently used for genetic transformation and other analyses. The calli were grown on MS medium supplemented with 0.5 mg l^−1^ IAA and 1.5 mg l^−1^ 6-BA at 25 °C in the dark. The calli were subcultured three times at 15 d intervals prior to use for genetic transformation and other assays.

Finally, rooted apple plantlets and tomatoes (*Solanum lycopersicum*) were transferred to pots containing a mixture of sand/perlite (1:1) and grown in a greenhouse under a 16 h/8 h light/dark and 25 °C day/night cycle. For the CdCl_2_ treatment, 3-month-old apple plantlets and tomatoes were cultivated in pots with 200 µM CdCl_2_ added for 2 weeks.

### Construction of expression vectors and genetic transformation

To construct sense overexpression vectors, sense full-length MdALMT14 cDNA was amplified. The resulting PCR products were inserted into the pCXSN vector under the control of the 35S promoter. The vector was introduced into ‘Orin’ calli and apple culture seedlings using *Agrobacterium tumefaciens* strain GV4404-mediated transformation. All primers used in the present study are listed in Supplementary Table S1 at *JXB* online.

### RNA extraction and quantitative real time PCR assays

Total fruit RNA was extracted using the hot borate method as previously described (Yao *et al.*, 2007). Total RNA from other tissues was extracted with Trizol reagent (Thermo Fisher Scientific, Waltham, MA, USA). Two micrograms of total RNA was used to synthesize first-strand cDNA with a PrimeScript First Strand cDNA Synthesis Kit (TaKaRa, Dalian, China).

For quantitative real time (qRT)-PCR analysis, the reactions were performed with iQ SYBR Green Supermix in an iCycler iQ5 system (Bio-Rad, Hercules, CA, USA) according to the manufacturer’s instructions. The relative quantification of specific mRNA levels was performed using the cycle threshold (*C*_t_) $2_{}^{-\Delta \Delta C_{t}}$ method (Software IQ5 2.0). For all analyses, the signal obtained for a gene of interest was normalized against the signal obtained for the 18S rRNA gene. All samples were analysed in three biological replicates.

### Protein extraction and western blot analysis

Approximately 500 mg of apple calli and culture seedlings were ground in a buffer containing 100 mM Tris (pH 8.0), 1 mM EDTA, 0.1 % (w/v) polyvinylpyrrolidone, 10 mM β-mercaptoethanol, 200 mM sucrose and 0.5 % (w/v) protease inhibitor mixture (Sigma-Aldrich, St Louis, MO, USA). After homogenization, the mixture was centrifuged (12 000 *g*, 10 min), and the protein concentration of the supernatant was determined using Bradford reagent (Sigma-Aldrich) with bovine serum albumin as a standard.

Anti-Myc monoclonal antibodies were prepared by the GenScript Company (Nanjing, China) and used to measure Myc-tagged MdALMT14 protein levels for western-blot analysis. Protein extracts from apple were separated by 12% SDS-PAGE and transferred to polyvinylidene fluoride membranes (Roche, Indianapolis, IN, USA) using an electrotransfer apparatus (Bio-Rad). The membranes were incubated with Myc or pMdALMT14^S358^ primary antibodies and then with peroxidase-conjugated secondary antibodies (Abcam, Shanghai, China), followed by visualization of immunoreactive proteins using of an ECL detection kit (Millipore, Billerica, MA, USA). Actin abundance served as a protein loading control.

### Co-immunoprecipitation procedures

For co-immunoprecipitation (Co-IP) assays, 1 mg of freshly extracted protein was pre-treated with 30 µl of Protein A/G agarose beads (4 h, 4 °C). The beads were centrifuged (1000 *g*, 5 min), and the supernatant was transferred into a fresh tube and incubated with Myc antibody (overnight, 4 °C). Next, 30 µl of protein A/G agarose beads was added to the supernatant (1 h, 4 °C) and after brief centrifugation (1000 *g*, 5 min) and four washing steps, loading buffer was added to the precipitates and the samples were boiled. For the Co-IP studies, the precipitates were further analysed by SDS-PAGE and western-blot analysis using standard procedures.

### Pull-down analysis

The full-length of MdSOS2L1 was amplified by PCR adding restriction enzyme sites and inserted into the *Eco*RI–*Sal*I sites of the pET-32a vector to produce a His-tagged recombinant protein. The full-length of MdALMT14 or MdALMT14^S358A^ coding region was cloned into the *Eco*RI–*Sal*I sites of the pGEX-4T-1 vector to produce sequence encoding a glutathione *S*-transferase (GST) fusion protein. For recombinant protein expression, the plasmids were transformed into *Escherichia coli* BL21 (DE3) cells (Transgene, Beijing, China), which were then cultured and induced with 0.1 mM isopropyl β-D-1-thiogalactopyranoside (IPTG) in Luria–Bertani (LB) broth for 6 h at 16 °C. For pull-down analysis with the GST- and His-tagged proteins, MdALMT14–GST or MdALMT14^S358A^–GST proteins were eluted from glutathione–agarose beads prior to incubation with MdSOS2L1–His attached to the tetradentate-chelated nickel resin. The protein samples were incubated for at least 4 h at 4 °C while being shaken, before centrifugation (1200 *g*, 2 min). The precipitates were washed at least three times to remove non-specific binding, followed by boiling (10 min, 100 °C).

### Cell-free degradation

Cells (*E. coli*, BL21) were induced with 0.1 mM IPTG and cultivated for 12 h at 16 °C. MdALMT14–GST protein was eluted from tetradentate-chelated nickel resin. Protein extracts were then obtained from the transgenic apple calli by homogenization degradation buffer containing 25 mM Tris–HCl, pH 7.5, 10 mM NaCl, 10 mM MgCl_2_, 4 mM phenylmethylsulfonyl fluoride, 5 mM dithiothreitol, and 10 mM ATP. The supernatant was collected, and the protein concentration was determined using Bradford assay reagent (Bio-Rad). Each reaction mixture contained 100 ng of MdALMT14–GST and 500 µg of protein extract from transgenic apple calli. For the proteasome inhibitor experiments, 20 µM MG132 was added 30 min prior to the experiment. The reactions were incubated at 22 °C and subsequently stopped by the addition of SDS-PAGE sample buffer and boiling (10 min, 100 °C). The results were quantified using Quantity One 1-D Analysis software (Bio-Rad).

### Construction of viral vectors and transient expression

To construct antisense viral expression vectors, an MdSOS2L1 DNA fragment was amplified using PCR and apple cDNA from shoot as a template. The PCR products were cloned into the tobacco rattle virus (TRV) vector in the antisense orientation under the control of the dual 35S promoter and the resulting vector was named MdSOS2L1-TRV. For transient expression studies, the vectors were transformed into *A. tumefaciens* for inoculation. The apple calli and leaf infections were performed as previously described (Hu *et al.*, 2016b). The injected apple leaves were soaked in 0 and 100 µM CdCl_2_ medium and kept in the light at room temperature for 1 h.

### DUAL membrane system analysis

The DUAL membrane system (Thaminy *et al.*, 2003) takes advantage of the split-ubiquitin mechanism to measure the interaction between an integral membrane protein and its interaction partners. MdSOS2L1 was fused to a mutated N-terminal half of ubiquitin (NubG), and a cDNA library was fused to the C-terminal half of ubiquitin (Cub) and the artificial transcription factor LexA-VP16. When the bait and prey interacting through NubG and Cub are forced into close proximity, these interactions result in the reconstitution of the split-ubiquitin. Split-ubiquitin is immediately recognized by ubiquitin-specific proteases, which then cleave the polypeptide chain between Cub and LexA-VP16. As a result, the artificial transcription factor is released from the membrane and translocated to the nucleus where it binds to the LexA operators situated upstream of a reporter gene via its LexA DNA binding domain. The VP16 transactivator domain then recruits the RNA polymerase II complex to the transcriptional start point of the reporter gene, resulting in its transcriptional activation and the ability of yeast to grow on defined minimal medium lacking histidine or adenine, and *lacZ*, encoding the enzyme β-galactosidase.

### Bimolecular fluorescence complementation assay

MdSOS2L1–YFPn and MdALMT14–YFPc were generated and used for transient expression in *Nicotiana benthamiana*. The constructs were transformed into *Agrobacterium* strain GV3101 using the freeze–thaw method. Cultured cells were harvested, resuspended in 10 mM MgCl_2_ plus 150 mM acetosyringone (Sigma-Aldrich), and then maintained at 25 °C for at least 3 h without shaking. *Agrobacterium* suspensions were infiltrated into *N. benthamiana* leaves with a needleless syringe. Leaf cells were analysed 2–3 d after infiltration.

### Fluorescence labelling of cadmium

Plant roots were stained using the Cd-specific probe Leadmium™ Green AM solution (Thermo Fisher Scientific). The methods are described in more detail in Shi *et al.* (2016).

## Results

### *MdSOS2L1* overexpressing plants showed improved Cd tolerance and increased malate accumulation/excretion

**Fig. 1.**
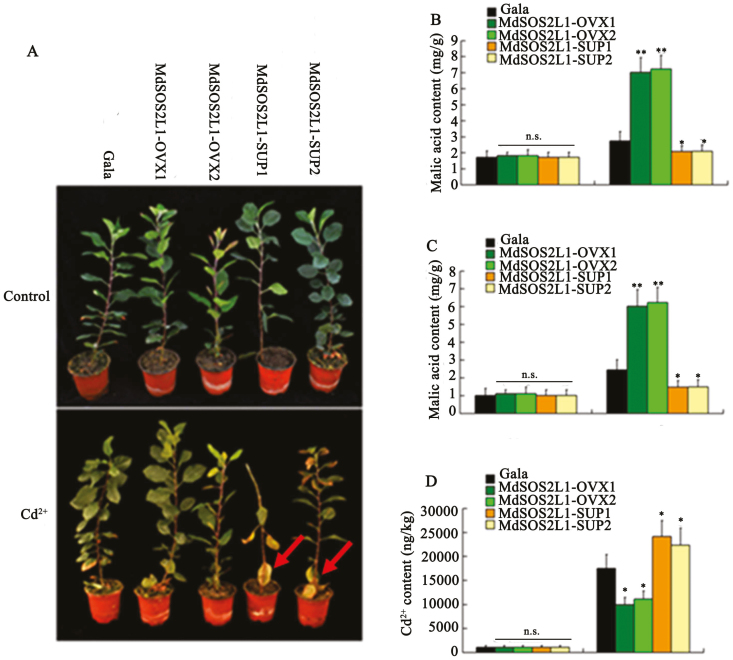
MdSOS2L1 overexpression increased cadmium (Cd) tolerance in transgenic apple plants. (A) Images of the two overexpression lines MdSOS2L1-OVX1 and 2, the two suppression lines MdSOS2L1-SUP1 and 2, and WT ‘Gala’ control plants with or without Cd^2+^ treatment. (B) Malate acid content in roots of the four transgenic lines and the WT ‘Gala’ control with or without Cd^2+^ treatment. (C) Excreted malate of the four transgenic lines and the WT ‘Gala’ control with or without Cd^2+^ treatment. (D) Cd^2+^ levels in the four transgenic lines and WT ‘Gala’ control with or without Cd^2+^ treatment. Error bars represent SD. Significance was assessed with a *t*-test: n.s., *P*>0.01; *P<0.01; **P<0.001.

Expression analysis indicated that *MdSOS2L1* transcript levels were induced by Cd^2+^ (Supplementary Fig. S1). To test if MdSOS2L1 had an effect on Cd tolerance, the ‘Gala’ control and *MdSOS2L1* transgenic soil-grown apple plants were subjected to Cd^2+^ stress. Apple plants that had been grown under normal conditions were used as controls. After treatment with 200 µM CdCl_2_ for 2 weeks, two *MdSOS2L1* overexpression lines, MdSOS2L1-OVX1 and MdSOS2L1-OVX2 (previously named as MdSOS2L1-S1 and MdSOS2L1-S2), showed a higher tolerance, while the two suppression lines, MdSOS2L1-SUP1 and MdSOS2L1-SUP2 (previously named as MdSOS2L1-AS1 and MdSOS2L1-AS6, 30), showed a lower tolerance than the WT control plants (Fig. 1A). Since malate is an organic acid that is important for responses to heavy metal stress (Bai *et al.*, 2012), we examined whether MdSOS2L1 influences malate accumulation by and excretion from roots in response to Cd^2+^ stress. The content of malic acid as well as the secreted malic acid was significantly increased in MdSOS2L1-overexpressing transgenic apple lines (MdSOS2L1-OVX1 and MdSOS2L1-OVX2), but decreased in MdSOS2L1 suppression lines (MdSOS2L1-SUP1 and MdSOS2L1-SUP2) compared with WT (Fig. 1B, C). The Cd^2+^ content was much lower in MdSOS2L1-OVX1 and MdSOS2L1-OVX2 roots and much higher in MdSOS2L1-SUP1 and MdSOS2L1-SUP2 roots than that in the WT control roots (Fig. 1D).

Ectopic expression of MdSOS2L1 in Arabidopsis and tomato (*S. lycopersicum*) increased Cd^2+^ tolerance and decreased Cd^2+^ accumulation in the tomato fruit (Supplementary Figs S2, S3). These results indicated that *MdSOS2L1* enhances Cd^2+^ tolerance by reducing its uptake and promoting malate accumulation/excretion.

### MdSOS2L1 interacted with MdALMT14

To determine how MdSOS2L1 influences the excretion of malate from the roots, a DUAL membrane system was used to screen an apple cDNA library for MdSOS2L1-interacting proteins. A truncated aluminum-activated malate transporter (ALMT) was identified as a positive clone in our yeast screen using MdSOS2L1 as bait. Using Arabidopsis ALMT1 as a query, a BLAST search was conducted of the apple genome database (The Apple Gene Function & Gene Family DataBase v1.0), and a total of 11 putative ALMT proteins were identified. A phylogenetic analysis indicated that the putative MdSOS2L1-interacting ALMT was closely related to AtALMT14, and it is hereafter referred to as MdALMT14 (Supplementary Fig. S4). Predictions of transmembrane structures using the TMHMM software (www.cbs.dtu.dk/services/TMHMM/) indicated that MdALMT14 had six transmembrane domains (Supplementary Fig. S5).

**Fig. 2.**
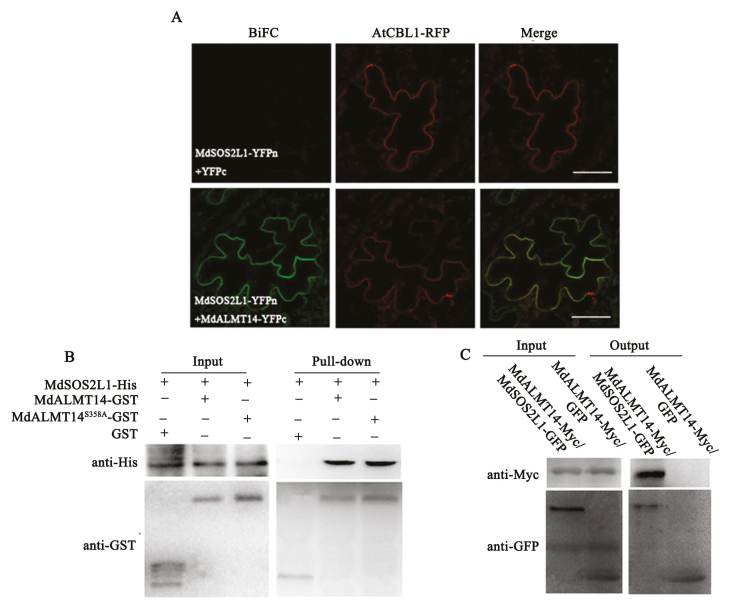
MdSOS2L1 interacted with MdALMT14. (A) BiFC was conducted in *Nicotiana benthamiana* to test the interaction between MdSOS2L1 and MdALMT14, with a plasma membrane marker (AtCBL1–red fluorescent protein (RFP)) included. (Scale bars: 100 µm.) (B) *In vitro* GST pull-down assays. MdSOS2L1–His proteins were incubated with immobilized MdALMT14–GST or GST, and the proteins immunoprecipitated with GST-beads were detected using anti-His antibody. (C) *In vivo* Co-IP assays between MdSOS2L1 and MdALMT14. Proteins were extracted from 35S::MdALMT14-Myc+35S::MdSOS2L1-GFP or 35S::MdALMT14-Myc+35S::GFP co-expressed in apple calli and immunoprecipitated with an anti-GFP antibody. The proteins from crude lysates (input) and the immunoprecipitated proteins (output) were detected with an anti-Myc antibody and anti-GFP antibody.

The interaction between MdSOS2L1 and MdALMT14 was further suggested by bimolecular fluorescence complementation (BiFC) assays in *N. benthamiana* using agroinfiltration. We observed green fluorescence indicative of heterodimer formation in the plasma membrane (Fig. 2A; Lu *et al.*, 2019). *AtCBL1* was used as a plasma membrane localization marker gene. To verify this interaction *in vitro*, a pull-down assay was performed. A MdSOS2L1–His fusion protein was respectively incubated with a MdALMT14–GST fusion protein and GST alone, and a pull-down assay was conducted using a His antibody. The MdSOS2L1–His protein was immunoprecipitated by MdALMT14–GST but not by GST alone, indicating that MdSOS2L1 physically interacted with MdALMT14 *in vitro* (Fig. 2B). The interaction between MdSOS2L1 and MdALMT14 was further confirmed by co-immunoprecipitation (Co-IP) assays using two types of double transgenic apple calli, i.e. 35S::MdALMT14-Myc/35S::green fluorescent protein (GFP) and 35S::MdALMT14-Myc/35S::MdSOS2L1-GFP. 35S::MdALMT14-Myc was immunoprecipitated with 35S::MdSOS2L1-GFP but not with GFP alone, indicating that MdSOS2L1 interacted with MdALMT14 *in vivo* (Fig. 2C).

### MdALMT14 overexpression increased malic acid excretion and Cd tolerance in transgenic apple plants

**Fig. 3.**
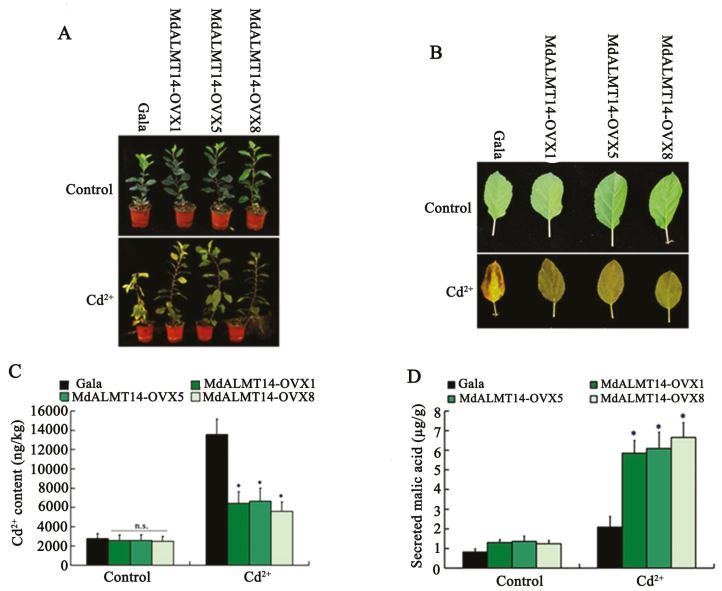
*MdALMT14* overexpression in transgenic apple plants increased the excretion of malate and enhanced Cd^2+^ tolerance. (A, B) Images of transgenic MdALMT14-OVX1, MdALMT14-OVX5, and MdALMT14-OVX8 plants and leaves grown with or without Cd^2+^ stress. (C, D) The Cd^2+^ content and the excretion of malic acid in the roots of transgenic MdALMT14-OVX1, MdALMT14-OVX5, and MdALMT14-OVX8 plants grown with or without Cd^2+^ stress. Error bars represent SD. Significance was assessed with a *t*-test: n.s., *P*>0.01; *P<0.01.

To characterize the function of *MdALMT14 in planta*, a constitutive overexpression vector, *35S::MdALMT14-Myc*, was constructed and transformed into the apple ‘Gala’ cultivar. Ten independent transgenic lines were obtained (Supplementary Fig. S6), of which three, MdALMT14-OVX1, MdALMT14-OVX5, and MdALMT14-OVX8, were selected for Cd^2+^ stress treatment. The three *MdALMT14* transgenic plants were grown in soil containing 200 µM CdCl_2_ for 2 weeks and the MdALMT14-overexpressing lines were much more tolerant of the CdCl_2_ treatment than the WT control (Fig. 3A, B). The Cd^2+^ content in the root of MdALMT14-OVX1, MdALMT14-OVX5, and MdALMT14-OVX8 were much lower than in root of the WT control (Fig. 3C).

Moreover, we detected the malate content and observed that the three transgenic lines excreted malate from the roots at almost the same level as the WT control under control conditions, but at a much higher level than the WT control when exposed to a Cd^2+^ treatment (Fig. 3D). Finally, these results indicated that *MdALMT14* overexpression promoted malic acid excretion from the roots, thereby improving Cd tolerance.

### Cd^2+^ induced phosphorylation of the MdALMT14 protein at Ser^358^

**Fig. 4.**
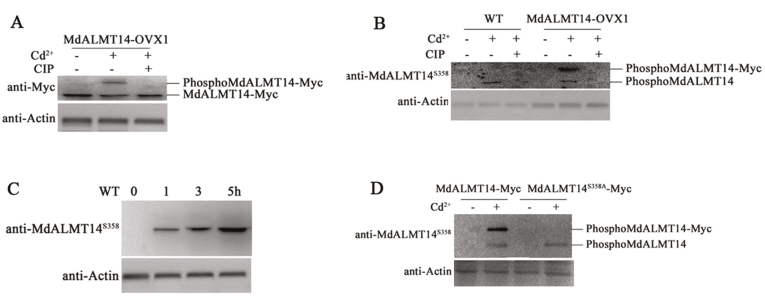
Cd^2+^ induced phosphorylation of the MdALMT14 protein. (A) Gel-shift assay of the MdALMT14 protein in the transgenic apple MdALMT14-OVX1 line using an anti-Myc antibody. Plants were treated with Cd^2+^ or with CIP for 1 h. (B) Western blot analysis detecting phosphorylation of the MALMT14 protein in the WT and transgenic MdALMT14-OVX1 plants using a specific anti-MdALMT14^S358^ antibody. (C) Cd^2+^-induced phosphorylation of the MdALMT14 protein increased with treatment time increased. WT ‘Gala’ apple plants were treated for different times (0, 1, 3, or 5 h). (D) Cd^2+^-induced phosphorylation of the MdALMT14 protein was absent in MdALMT14^S358A^–Myc transgenic apple calli using the anti-MdALMT14^S358^ antibody. For western blot assays, actin was used as a loading control to ensure equal loading.

Protein extracts from transgenic MdALMT14-OVX1 plants that had been treated or not with Cd^2+^ were used in immunoblot analyses with an anti-Myc antibody. The apparent molecular mass of the MdALMT14–Myc proteins was higher in transgenic plants treated with Cd^2+^ compared with those grown without Cd^2+^, indicating that Cd^2+^ potentially induced a post-translational modification of the MdALMT14 protein. Furthermore, treatment of the extracts with calf intestine alkaline phosphatase (CIP) converted the immunoreactive MdALMT14 to a lower molecular mass form, indicating that the Cd^2+^-induced post-translational modification of MdALMT14 was predominantly phosphorylation (Fig. 4A).

To identify potential phosphorylation sites in the MdALMT14 protein, the more slowly migrating immunoreactive proteins were captured with anti-Myc antibody-conjugated agarose beads and separated using SDS-PAGE. After proteolytic digestion and purification, the protein sample was analysed by liquid chromatography–tandem mass spectrometry. This indicated a serine residue at position 358 (Ser^358^) as a potential phosphorylation site in MdALMT14 following Cd^2+^ treatment (Supplementary Fig. S7).

We then prepared an anti-MdALMT14^S358^ monoclonal antibody to detect Ser^358^-phosphorylated MdALMT14 protein. Immunoblot assays were conducted with the anti-MdALMT14^S358^ antibody and protein extracts from MdALMT14-OVX1 plants and the WT control with or without Cd^2+^ stress. The antibody recognized Cd-induced phosphorylated MdALMT14 proteins in the WT extracts, and both the phosphorylated MdALMT14 and MdALMT14–Myc isoforms in transgenic plants, indicating that Cd^2+^ induced phosphorylation of the MdALMT14 and MdALMT14–Myc proteins at Ser^358^ (Fig. 4B).

In addition, WT plants were treated with Cd^2+^ for 0, 1, 3, and 5 h to examine whether treatment time influenced MdALMT14 phosphorylation. We observed that the phosphorylation intensity of the MdALMT14 protein gradually increased with treatment duration (Fig. 4C), indicating that MdALMT14 was phosphorylated in response to Cd^2+^ and that this modification was positively associated with Cd^2+^ treatment time.

To determine whether the Ser^358^ residue is required for the Cd^2+^-induced phosphorylation of the MdALMT14 protein, a mutated form was generated with Ser^358^ replaced by Ala^358^. The mutated MdALMT14^S358A^ sequence was inserted downstream of the *35S* promoter, and the resulting construct (35S::MdALMT14^S358A^-Myc) was transformed into apple calli from the ‘Orin’ cultivar. The transgenic calli were then used in immunoblot assays with the anti-MdALMT14^S358^ antibody to determine whether Ser^358^ is crucial for Cd^2+^-induced phosphorylation of the MdALMT14 protein. We observed that the mutation from Ser^358^ to Ala^358^ completely abolished the phosphorylation modification (Fig. 4D), indicating a key role for this residue.

### MdSOS2L1 was required for Cd^2+^-induced phosphorylation of the MdALMT14 protein at Ser^358^

**Fig. 5.**
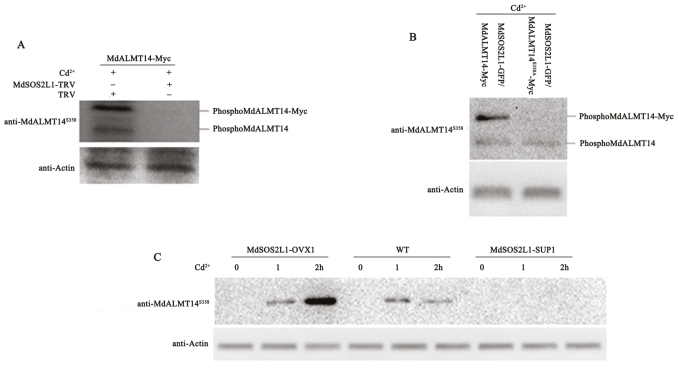
MdSOS2L1 was required for Cd^2+^-induced phosphorylation of the MdALMT14 protein. (A) MdALMT14 phosphorylation was examined in 35S::MdALMT14-Myc transgenic apple calli, which were infected or not with the virus-based vector MdSOS2L1-TRV after Cd^2+^ treatment for 1 h. TRV empty vector-infected apple calli were used as the control. Western blot analyses were performed with an anti-MdALMT14^S358^ antibody. (B) MdALMT14 phosphorylation in Cd^2+^-treated MdSOS2L1–GFP/MdALMT14–Myc and MdSOS2L1–GFP/MdALMT14^S358A^–Myc calli, using an anti-MdALMT14^S358^ antibody. (C) Western blot analysis using the anti-MdALMT14^S358^ antibody showed the MdALMT14 phosphorylation in two overexpression lines, MdSOS2L1-OVX1 and -2, two suppression lines, MdSOS2L1-SUP1 and -2, and WT control with or without Cd^2+^ treatment.

To investigate whether MdSOS2L1 is required for Cd^2+^-induced phosphorylation of the MdALMT14 protein, we used a tobacco rattle virus (TRV) viral vector to down-regulate *MdSOS2L1* expression in transgenic 35S::MdALMT14-Myc calli. Both 35S::MdALMT14-Myc+TRV and 35S::MdALMT14-Myc+MdSOS2L1-TRV transgenic calli were treated with Cd^2+^ and used as a source of proteins for immoblot assays with the anti-MdALMT14^S358^ antibody. We observed that Cd^2+^-induced phosphorylation of both MdALMT14 and MdALMT14-Myc was abolished when MdSOS2L1 expression was suppressed (Fig. 5A), indicating that the MdSOS2L1 protein kinase was required for Cd^2+^-induced phosphorylation of the MdALMT14 protein.

The 35S::MdSOS2L1-GFP expression vector was then introduced into 35S::MdALMT14-Myc and 35S::MdALMT14^S358A^-Myc transgenic calli, resulting in double transgenic calli, to examine Cd^2+^-induced phosphorylation with the phospho-specific anti-MdALMT14^S358^ antibody. The results suggested that both MdALMT14 and MdALMT14–Myc were phosphorylated in the double transgenic MdALMT14–Myc/MdSOS2L1–GFP calli, while only MdALMT14–Myc and not MdALMT14^S358A^–Myc was phosphorylated in the double transgenic MdALMT14^S358A^–Myc/MdSOS2L1–GFP calli (Fig. 5B). These results indicated that Ser^358^ is required for MdSOS2L1-mediated phosphorylation of the MdALMT14 protein in response to Cd^2+^ stress.

Finally, MdSOS2L1-OVX1, MdSOS2L1-SUP1, and WT plants were treated with Cd^2+^ and used in phosphorylation assays with the phospho-specific anti-MdALMT14^S358^ antibody. The phosphorylation intensity of the MdALMT14 protein was much stronger in MdSOS2L1-OVX1 plants than in the WT control (Fig. 5C). In contrast, there was almost no phosphorylation signal from plants of the suppression line MdSOS2L1-SUP1 (Fig. 5C), suggesting that MdSOS2L1 is required for Cd^2+^-induced phosphorylation of MdALMT14.

### MdSOS2L1 enhanced the stability of MdALMT14

**Fig. 6.**
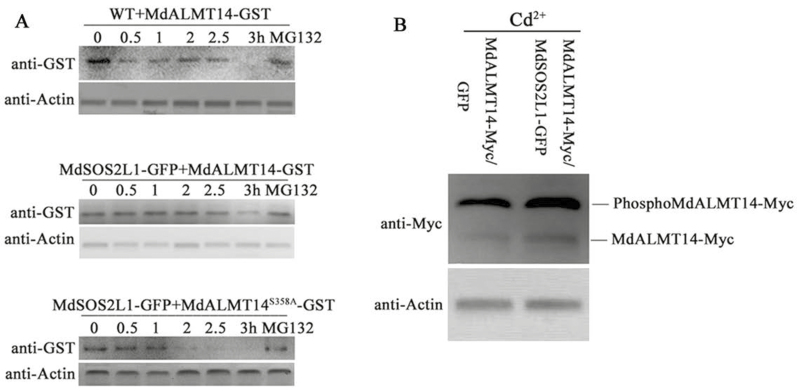
MdSOS2L1-mediated phosphorylation stabilized the MdALMT14 protein. (A) Cell-free degradation assay of recombinant MdALMT14–GST or MdALMT14^S358A^–GST protein in the protein extracts of transgenic apple calli, as labeled. Actin abundance was used as a loading control to ensure equal loading. Protein levels of MdALMT14–GST and MdALMT14^S358A^–GST were visualized by immunoblotting with an anti-GST antibody. (B) MdALMT14 abundance in MdALMT14–Myc+GFP and MdALMT14–Myc+MdSOS2L1–GFP transgenic calli treated with Cd^2+^ stress.

To determine whether MdSOS2L1 influenced MdALMT14 stability, an *in vitro* cell-free protein degradation assay was performed. Recombinant MdALMT14–GST and MdALMT14^S358A^–GST proteins were expressed in, and purified from, *E. coli*, and then incubated with protein sample extracted from MdSOS2L1-OVX1 and WT plants. Protein gel blots showed that MdALMT14–GST recombinant proteins degraded more slowly in extracts from MdSOS2L1-OVX1 plants than in those from the WT control. Additionally, MdALMT14^S358A^–GST degraded much more rapidly than MdALMT14–GST proteins (Fig. 6A). Additionally, the presence of MG132 markedly inhibited the degradation of MdALMT14–GST and MdALMT14^S358A^–GST (Fig. 6A), suggesting that MdALMT14 is likely degraded via the 26S proteasome, and that MdSOS2L1-mediated MdALMT14 phosphorylation at Ser^358^ inhibited this process.

When the abundance of MdALMT14–Myc was examined with an anti-Myc antibody in MdALMT14–Myc+GFP and MdALMT14–Myc+MdSOS2L1–GFP transgenic calli under Cd^2+^ stress conditions, we saw that the 35S::MdALMT14-Myc+35S::MdSOS2L1-GFP calli accumulated higher levels of MdALMT14–Myc than the 35S::MdALMT14-Myc+35S::GFP calli (Fig. 6B), indicating that MdSOS2L1 enhanced the stability of MdALMT14 *in vitro* and promoted its accumulation *in vivo*.

### MdSOS2L1 enhanced Cd^2+^ tolerance in an MdALMT14-dependent manner

**Fig. 7.**
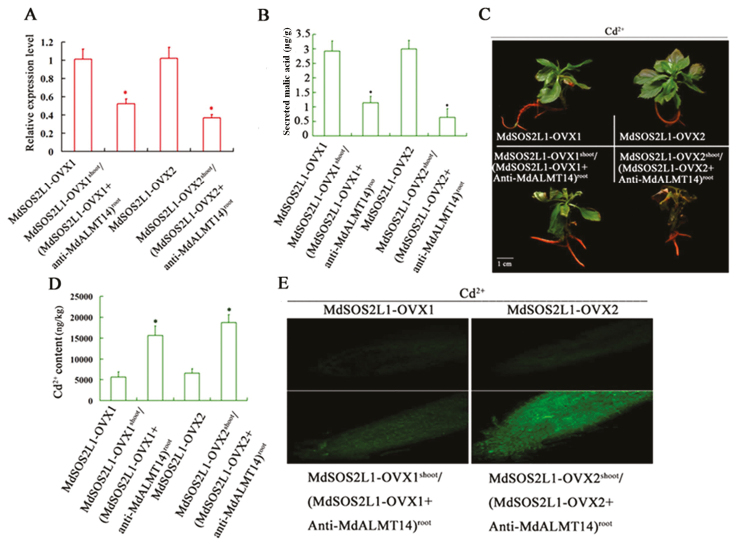
MdSOS2L1 increased the excretion of malate and Cd^2+^ tolerance in an MdALMT14-dependent manner. (A) Expression levels of *MdALMT14* in MdSOS2L1-OVX1, MdSOS2L1-OVX1^shoot^/(MdSOS2L1-OVX1+anti-MdALMT14)^root^, MdSOS2L1-OVX2, and MdSOS2L1-OVX2^shoot^/(MdSOS2L1-OVX2+anti-MdALMT14)^root^ transgenic plants. (B) The excretion of malic acid from the roots of MdSOS2L1-OVX1, MdSOS2L1-OVX1^shoot^/(MdSOS2L1-OVX1+anti-MdALMT14)^root^, MdSOS2L1-OVX2, and MdSOS2L1-OVX2^shoot^/(MdSOS2L1-OVX2+anti-MdALMT14)^root^ transgenic plants grown under Cd^2+^ stress. (C) Cd tolerance of MdSOS2L1-OVX1, MdSOS2L1-OVX1^shoot^/(MdSOS2L1-OVX1+anti-MdALMT14)^root^, MdSOS2L1-OVX2, and MdSOS2L1-OVX2^shoot^/(MdSOS2L1-OVX2+anti-MdALMT14)^root^ transgenic plants grown under Cd^2+^ stress. (D) Cd^2+^ content in the roots of MdSOS2L1-OVX1, MdSOS2L1-OVX1^shoot^/(MdSOS2L1-OVX1+anti-MdALMT14)^root^, MdSOS2L1-OVX2, and MdSOS2L1-OVX2^shoot^/(MdSOS2L1-OVX2+anti-MdALMT14)^root^ transgenic plants grown under Cd^2+^ stress. (E) Leadmium^TM^ Green AM Dye showing Cd contents in the roots of MdSOS2L1-OVX1, MdSOS2L1-OVX1^shoot^/(MdSOS2L1-OVX1+anti-MdALMT14)^root^, MdSOS2L1-OVX2, and MdSOS2L1-OVX2^shoot^/(MdSOS2L1-OVX2+anti-MdALMT14)^root^ transgenic plants grown under Cd^2+^ stress. The values are presented as the mean of three replicates, and differences with a *P*-value<0.05 were considered significant. Error bars represent SD. Significance was assessed with a *t*-test: *P<0.01.

To examine whether MdALMT14 is required for MdSOS2L1 function, *MdALMT14* expression was specifically suppressed in the roots of the transgenic lines MdSOS2L1-OVX1 and MdSOS2L1-OVX2, using an *Agrobacterium rhizogenes*-mediated transformation method. *A. rhizogenes* strain MSU440, containing a specific MdALMT14 antisense cDNA fragment, was used to quickly induce regeneration of *MdALMT14*-down-regulated hairy roots. The successfully transformed roots were easily distinguished because they exogenously expressed red fluorescent protein (RFP). A strong RFP signal and reduced *MdALMT14* transcript levels were observed in the transformed roots, demonstrating that the *MdALMT14* antisense suppression vector was functional in the hairy roots (Supplementary Fig. S8; Fig. 7A). Two MdSOS2L1 transgenic lines and two co-expressing plants (MdSOS2L1-OVX1^shoot^/(MdSOS2L1-OVX1+Anti-MdALMT14)^root^ and MdSOS2L1-OVX2^shoot^/(MdSOS2L1-OVX2+Anti-MdALMT14)^root^) were then treated with Cd for 2 weeks. As a result, the two co-expressing MdSOS2L1-OVX1^shoot^/(MdSOS2L1-OVX1+Anti-MdALMT14)^root^ and MdSOS2L1-OVX2^shoot^/(MdSOS2L1-OVX2+Anti-MdALMT14)^root^ plants secreted less malate from the roots than the MdSOS2L1-OVX1 and MdSOS2L1-OVX2 transgenic plants (Fig. 7B). As a result, *MdALMT14* suppression in the roots reduced the Cd^2+^ tolerance of the MdSOS2L1 transgenic lines, as indicated by wrinkled leaves (Fig. 7C).

Staining with Leadmium^TM^ Green AM confirmed that less Cd^2+^ accumulated in MdSOS2L1-OVX1 and MdSOS2L1-OVX2 roots compared with MdSOS2L1-OVX1^shoot^/(MdSOS2L1-OVX1+Anti-MdALMT14)^root^ and MdSOS2L1-OVX2^shoot^/(MdSOS2L1-OVX2+Anti-MdALMT14)^root^ roots (Fig. 7D, E). This suggested that MdSOS2L1 promoted malate excretion out of the roots and enhanced Cd^2+^ resistance, at least partially, in an MdALMT14-dependent manner.

## Discussion

Cd is a non-essential heavy metal element that is highly toxic to almost all organisms, including plants and humans. In the present study, a protein kinase, MdSOS2L1, was shown to improve Cd tolerance in apple by interacting with and phosphorylating the malate transporter MdALMT14. This result indicated that MdSOS2L1 mediated the promotion of malate excretion from the roots and the enhancement of Cd tolerance. These findings provide new insights into the molecular mechanism by which plants respond to Cd stress and the promotion of Cd tolerance, at least partially, in a malate-dependent manner.

Protein kinase SOS2 promotes salt tolerance through the regulation of ion transport (Batelli *et al.*, 2007). SOS2 also phosphorylates VHA-B1 and VHA-B2 to modulate the activity of the vacuolar proton pump, V-ATPase. Vacuolar proton pumps, such as V-ATPase and V-PPase, are involved in the establishment and maintenance of the electrochemical potential of the tonoplast, which provides the driving force for the active transport of metabolites, such as malate, and heavy metals including Cd and Cd chelates (Gaxiola *et al.*, 2007; Shiratake & Martinoia, 2007; Hu *et al.*, 2016a). As a result, *MdSOS2L1*-overexpressing plants accumulated not only more malate, but also more Cd in the vacuole than does the WT control (Hu *et al.*, 2016a). Malate acts as a ligand and forms complexes with heavy metals and is known to play an important role in heavy metal detoxification through vacuolar sequestration (Baker and Proctor, 1990). Generally, endogenous malic acid levels increase with heavy metal stress in plants (Tolrà *et al.*, 1996; Shen *et al.*, 1997), and heavy metal-tolerant plants accumulate more organic acids than do sensitive plants (Godbold *et al.*, 1984). The AttDT (tonoplast dicarboxylate transporter) mutant accumulates less malate and exhibits decreased tolerance to heavy metals (Emmerlich *et al.*, 2003; Hurth *et al.*, 2005), and therefore, malate accumulation is conducive for enhancement of heavy metal tolerance in plants. MdSOS2L1 promotes this, at least partially, by modulating vacuolar proton pump activity, malate accumulation and vacuolar sequestration.

Cd stress disturbs plant antioxidant defenses and induces the production of reactive oxygen species (ROS) (Rodríguez-Serrano *et al.*, 2009; Fernández *et al.*, 2013). Intracellular malic acid is a core metabolite in plants cell and plays important roles in physiology and metabolism, acting as an osmoticum, a regulator of pH homeostasis, and an antioxidant metabolite (Naya *et al.*, 2007). In sunflower (*Helianthus annuus*), malate diminishes Cd toxicity by enhancing root activity and reducing H_2_O_2_ levels (Hawrylak-Nowak *et al.*, 2015), and since MdSOS2L1-overexpressing plants generated less H_2_O_2_ and O^2−^ than the WT, malic acid likely scavenges damaging ROS.

In addition to the high accumulation of malate in the vacuole, overexpressing plantlets also excreted more malate into the rhizosphere under Cd^2+^ stress than the WT control (Fig. 1). Cd-induced organic acid secretion from the roots helps exclude Cd and reduces Cd accumulation (Nigam *et al.*, 2001; Zhu *et al.*, 2011), and the reduction of Cd influx with supplementary organic acid may be ascribed to the formation of a metal–oxalate complex and a decrease in Cd bioavailability (Xu *et al.*, 2015; Guo *et al.*, 2017). The presence of citric acid was reported to alleviate the toxicity of Pb and Cd in radish (*Raphanus raphanistrum* subsp. *sativus*) by decreasing the adsorption of Cd, mainly due to a pH decrease (Chen *et al.*, 2016). Generally, Cd-tolerant plant species exhibit higher malate exudation rates than Cd-sensitive species under Cd stress (Guo *et al.*, 2017). In tomato, Cd-induced oxalate secretion from the root apex helps to exclude Cd from the roots, thus contributing to less Cd accumulation in Cd-tolerant than in Cd-sensitive cultivars (Zhu *et al.*, 2011). In the present study, *MdSOS2L1*-overexpressing transgenic plants accumulated less Cd, while *MdSOS2L1* suppression lines accumulated more Cd than the WT control (Fig. 1), indicating that *MdSOS2L1* promotes malate excretion and prevents Cd uptake. We conclude that MdSOS2L1 enhances the tolerance of apple to Cd (Fig. 1).

In the context of responses to abiotic stress, anion channels have been classified as slow ‘S-type’ (SLAC) and the rapid ‘R-type’ (AtALUMINUM ACTIVATED MALATE TRANSPORTERS/QUICK ANION CHANNEL (AtALMTs/QUAC)) anion efflux channels. Al-activated malate transporters (ALMTs) play a critical role in malate release and heavy metal stress responses (Sasaki *et al.*, 2004; Delhaize *et al.*, 2007; Kobayashi *et al.*, 2007), and they have been well studied in various plant species. The genes encoding ALMT proteins, such as *TaALMT1* and *AtALMT1*, have been identified from *Triticum aestivum* (Sasaki *et al.*, 2004) and Arabidopsis (Hoekenga *et al.*, 2006), and these proteins have been found to have a central role in Al resistance by releasing malate from the root tips, which chelates Al in the rhizosphere (Sasaki *et al.*, 2004; Hoekenga *et al.*, 2006). In maize (*Zea mays*), ZmALMT1 activates Al^3+^ independently, and transports inorganic anions, such as Cl^−^, NO_3_^−^, and SO_4_^2−^, rather than malate (Piñeros *et al.*, 2008). In Arabidopsis, AtALMT12 is strongly expressed in guard cells, and loss-of-function mutants are impaired in stomatal closure (Sasaki *et al.*, 2010). In the present study, an AtALMT14 homolog from apple, MdALMT14, was identified (Supplementary Fig. S4), and shown to regulate malate secretion following Cd^2+^ treatment, consistent with a role in Cd tolerance (Fig. 3).

Phosphorylation is associated with regulating the activity of anion channels. The SLAC1 anion channel is activated through direct phosphorylation by four calcium-independent kinases, including SnRK2 type kinase (sucrose nonfermenting-1 (Snf1)-related protein kinase 2) OST1, CPKs (Ca^2+^-dependent protein kinases), CIPKs (CBL-interacting protein kinases) and the receptor-like kinase GHR1 (Geiger *et al.*, 2009; Hua *et al.*, 2012; Maierhofer *et al.*, 2014; Lind *et al.*, 2015; Zou *et al.*, 2015). The activity of AtALMT12/QUAC1 is regulated in a phosphorylation-dependent manner by the Open Stomata 1 (OST1) kinase, which is activated by abscisic acid under stress conditions (Meyer *et al.*, 2010; Imes *et al.*, 2013). In the wheat channel TaALMT1, the amino acid Ser^384^ is a key residue for regulation of channel activity via direct protein phosphorylation (Ligaba *et al.*, 2009). In the present study, MdSOS2L1 interacted with, and phosphorylated, the MdALMT14 protein, enhancing its stability (Figs 2, 5, 6). These findings suggest that MdSOS2L1–MdALMT14 acts as a regulatory module responding to Cd and other heavy metals. This provides new insights into the molecular mechanisms by which plants secrete malate from roots into the rhizospheres in response to Cd and other heavy metals to enhance tolerance. A detailed examination of phosphorylation-dependent activation of evolutionarily diverse ALMT/QUAC channels would likely provide new mechanistic insights into kinase regulation of R-type anion currents.

Heavy metal pollution is a significant global environmental problem (DalCorso *et al.*, 2010; Luo *et al.*, 2018). It is therefore of great importance to further dissect the processes of heavy metal detoxification and signaling pathways in plants (Luo *et al.*, 2018). The findings of the present study shed light on the roles of the MdSOS2L1–MdALMT14 complex in physiological responses to Cd and other heavy metal toxicities in plants. These results may provide valuable information for molecular breeding and evolutionary studies. Increasing the current knowledge of the mechanisms that enable plants to tolerate heavy metal stress may help in developing new strategies for phytoremediation.

## Supplementary data

Supplementary data are available at *JXB* online.

Fig. S1. Cd^2+^ induced the expression of *MdSOS2L1*.

Fig. S2. Overexpression *MdSOS2L1* transgenic plants improved Cd^2+^ resistance in Arabidopsis.

Fig. S3. Overexpression *MdSOS2L1* transgenic plants improved Cd^2+^ resistance in tomato.

Fig. S4. The phylogenetic tree of MdALMT14 and AtALMTs.

Fig. S5. The transmembrane domains of MdALMT14.

Fig. S6. The expression of *MdALMT14* was detected in transgenic plants.

Fig. S7. Collision-induced dissociation mass spectrum showed the phosphorylation site was serine (S) at residue 358 (S358) of the MdALMT14 protein.

Fig. S8. RFP signal was observed in co-expressed plants MdSOS2L1-OVX1^shoot^/(MdSOS2L1-OVX1+Anti-MdALMT14)^root^ and MdSOS2L1-OVX2^shoot^/(MdSOS2L1- OVX2+Anti-MdALMT14)^root^.

Table S1. Primers used in this study.

eraa121_suppl_Supplementary_File_001
