# Supplementary material for: Phosphorylation of a malate transporter promotes malate excretion and reduces cadmium uptake in apple
Source: J Exp Bot. 2020 Mar 9;71(12):3437–49. doi: 10.1093/jxb/eraa121 (PMC7475249; doi:10.1093/jxb/eraa121)
Supplement: eraa121_suppl_Supplementary_File_001 [file eraa121_suppl_supplementary_file_001.pdf]

Figure S1. Cd^{2+} induced the expression of *MdSOS2L1*.

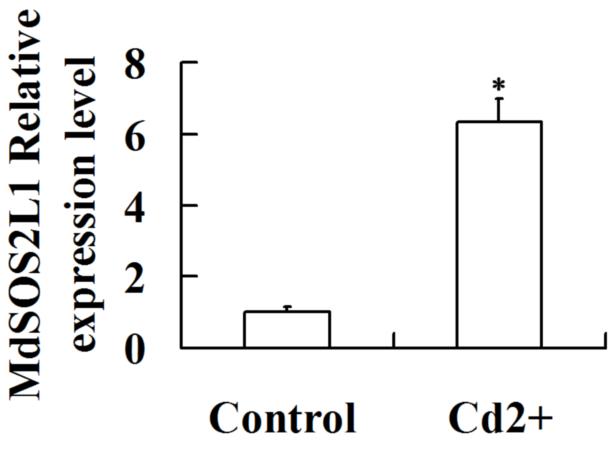

Figure S2. Overexpression *MdSOS2L1* transgenic plants improved Cd^{2+} resistance in *A. thaliana*. (A) The phenotype of plant lines Col, sos2-2, MdSOS2L1-1, 2 under $CdCl_2$ treatment. (B) root length; (C) the chlorophyll content; (D) the Cd^{2+} content

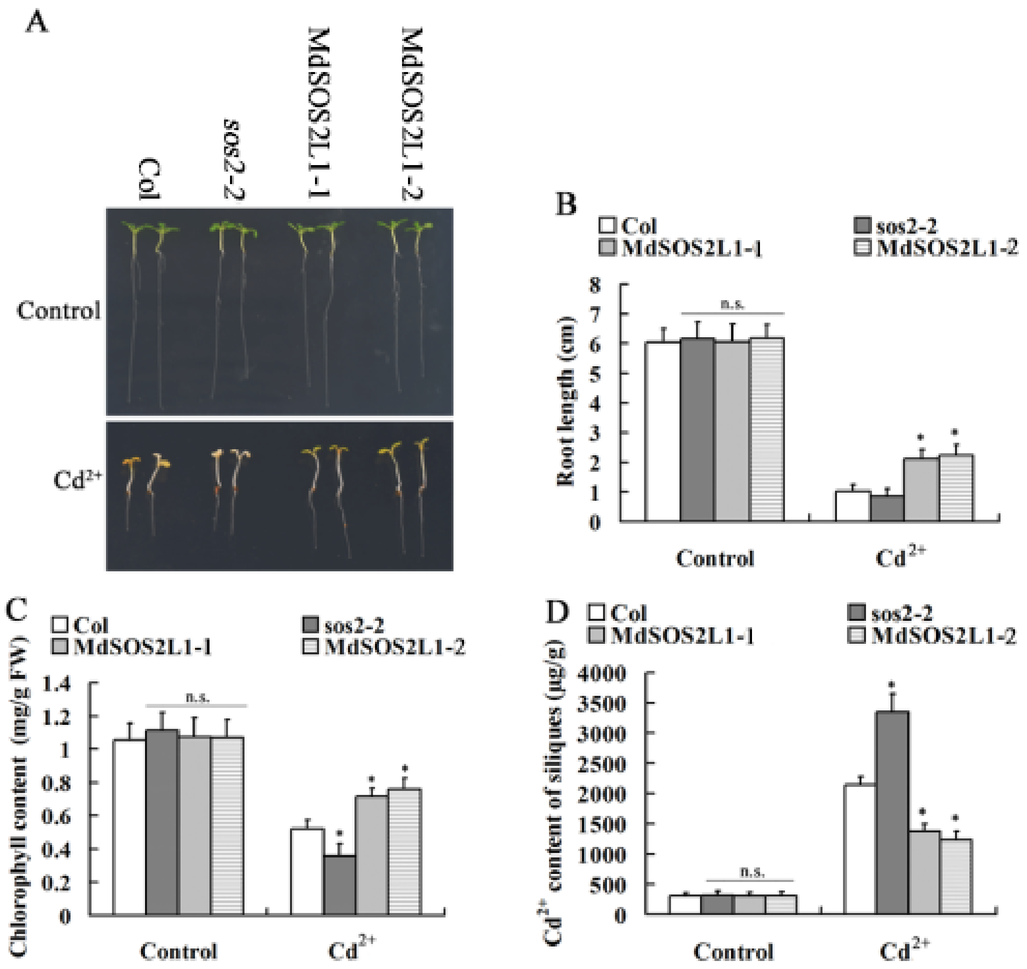

Figure S3. Overexpression *MdSOS2L1* transgenic plants improved Cd^{2+} resistance in tomato. (A) The phenotype of plant lines WT, MdSOS2L1-1 and MdSOS2L1-4 under $CdCl_2$ treatment. (B) plant height; (C) the Cd^{2+} content of fruit.

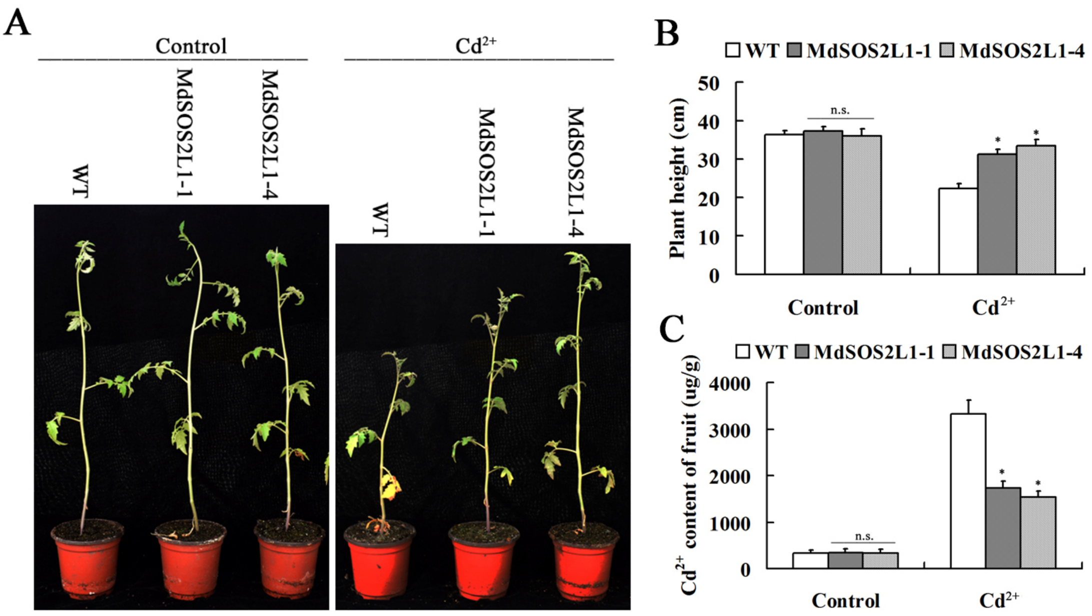

Figure S4. The phylogenetic tree of MdALMT14 and AtALMTs.

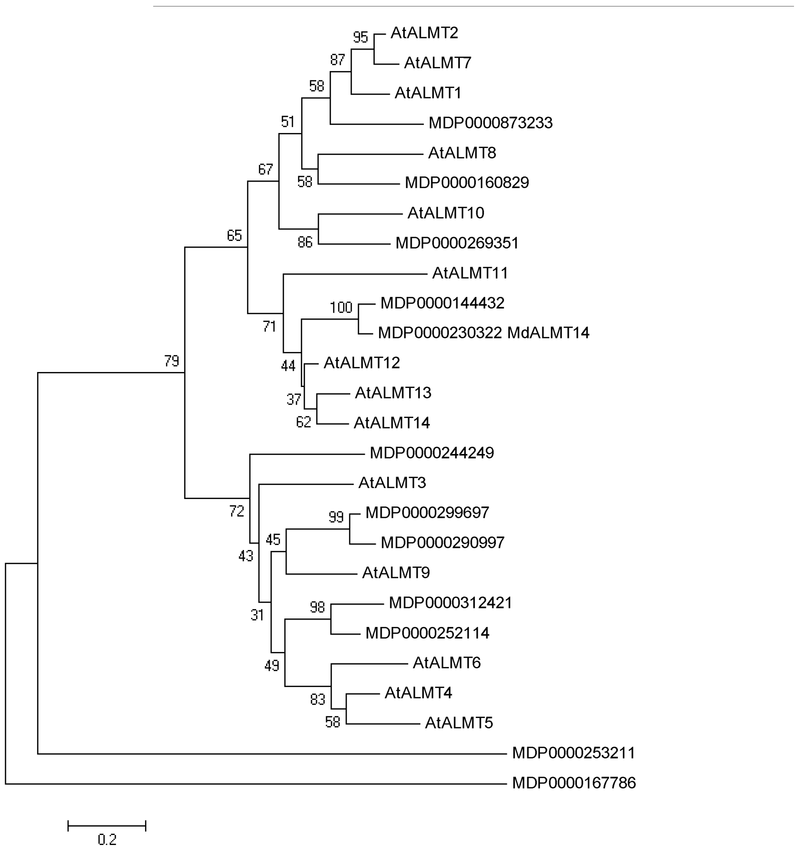

Figure S5. The transmembrane domains of MdALMT14.

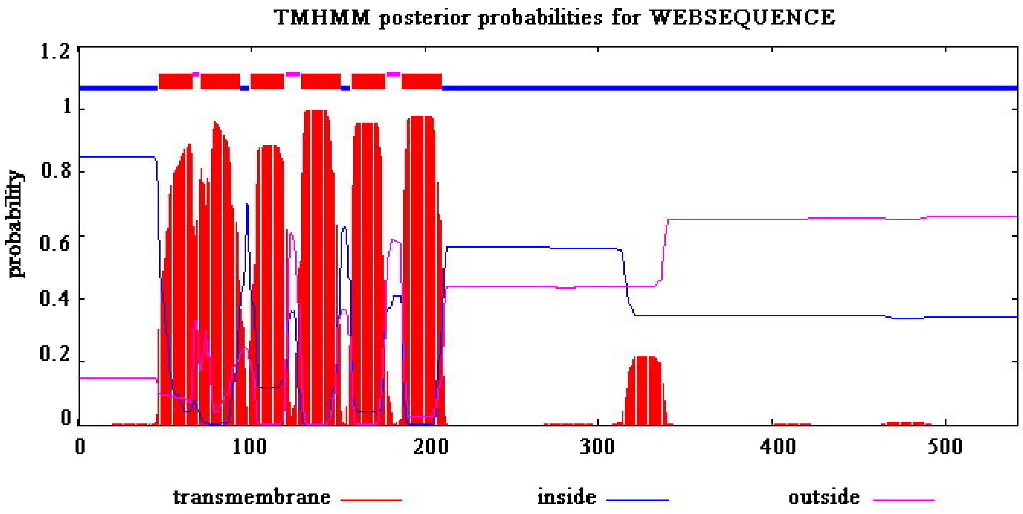

Figure S6. The expression of MdALMT14 was detected in transgenic plants.

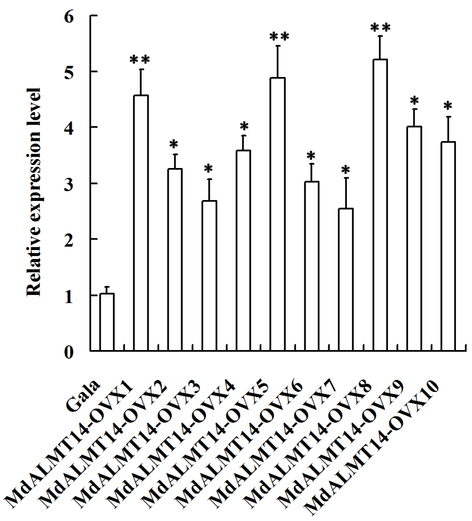

Figure S7. Collision-induced dissociation mass spectrum showed the phosphorylation site was serine (S) at residue 358 (S358) of the MdALMT14 protein.

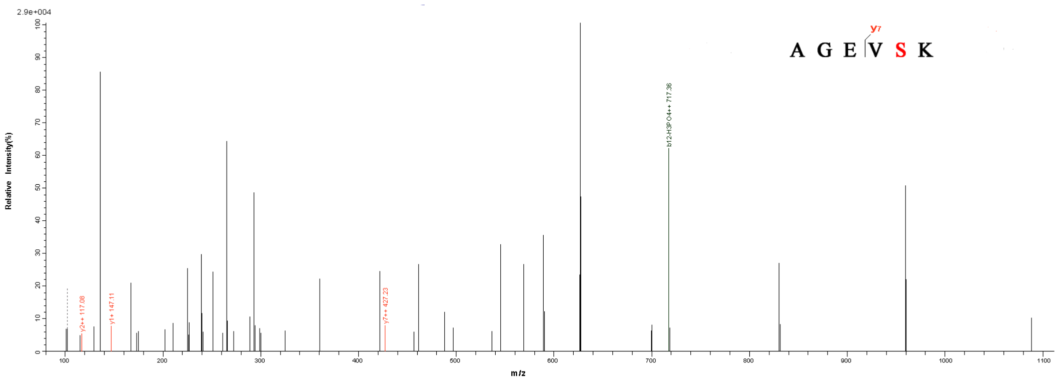

Figure S8. RFP signal was observed in co-expressed plants MdSOS2L1-OVX1^{shoot}/(MdSOS2L1-OVX1+Anti-MdALMT14)^{root} and MdSOS2L1-OVX2^{shoot}/(MdSOS2L1-OVX2+Anti-MdALMT14)^{root}.

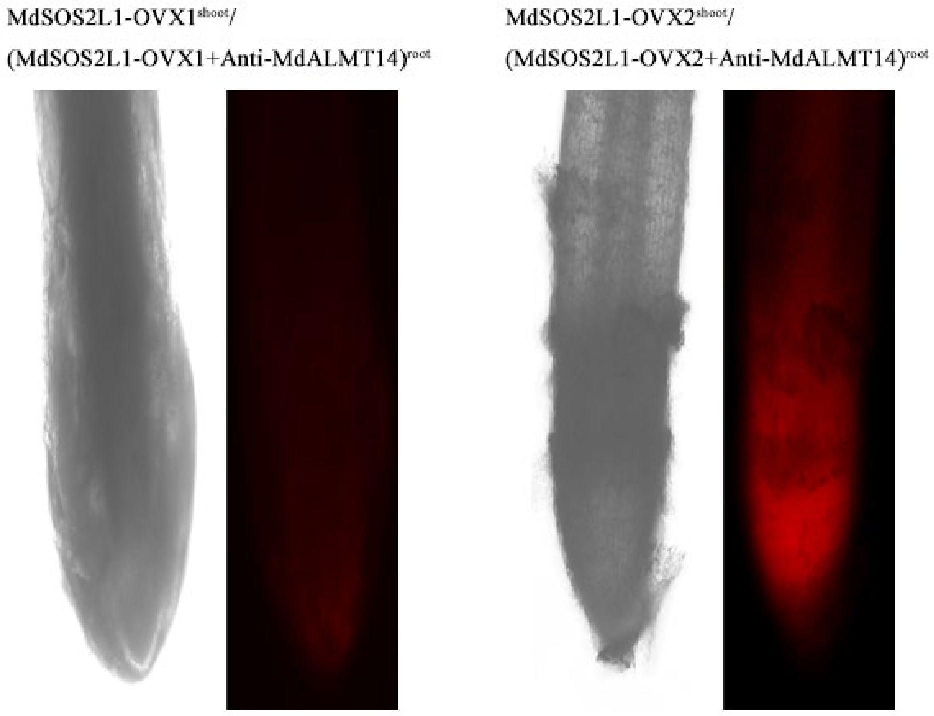

Table S1. Primers used in this study.

Primer	Sequence (5'to 3')
MdALMT14-F	GTTTCCCAAAGTTCATGCAGG
MdALMT14-R	CTTGCTTGTACAGATTAAGGC
qMdALMT14-F	GCTGACTCTTACCCCTTGTG
qMdALMT14-R	CTGGGAGTTTTTGTGTAGGGTAAT
ALMT14-Myc-F	AATGGACATGGGCAAGGAT
ALMT14-Myc-R	GTCGACTTAATCAGCTCCATG
ALMT14-GST-F	GAATTCATGGACATGGGCAAGG
ALMT14-GST-R	GTCGACTTAATCAGCTCCATGGG
18S -F	CACGGGGAGGTAGTGACAA
18S -R	CCTCCAATGGATCCTCGTTA
MdALMT14(MSU440)-F	GTCGACTGAATTCATGGACATGGGCAAG
MdALMT14 (MSU440)-R	CCCGGGATCAGCTCCATGGGAAG
